# Spontaneous Akt2 deficiency in a colony of NOD mice exhibiting early diabetes

**DOI:** 10.1038/s41598-024-60021-w

**Published:** 2024-04-20

**Authors:** Julie Hervé, Karine Haurogné, Marie Allard, Sophie Sourice, Pierre Lindenbaum, Jean-Marie Bach, Blandine Lieubeau

**Affiliations:** 1 Oniris, INRAE, IECM, Nantes, France; 2 Nantes Université, GenoBIRD, Nantes, France

**Keywords:** Physiology, Diseases, Endocrinology

## Abstract

Diabetes constitutes a major public health problem, with dramatic consequences for patients. Both genetic and environmental factors were shown to contribute to the different forms of the disease. The monogenic forms, found both in humans and in animal models, specially help to decipher the role of key genes in the physiopathology of the disease. Here, we describe the phenotype of early diabetes in a colony of NOD mice, with spontaneous invalidation of Akt2, that we called HYP. The HYP mice were characterised by a strong and chronic hyperglycaemia, beginning around the age of one month, especially in male mice. The phenotype was not the consequence of the acceleration of the autoimmune response, inherent to the NOD background. Interestingly, in HYP mice, we observed hyperinsulinemia before hyperglycaemia occurred. We did not find any difference in the pancreas’ architecture of the NOD and HYP mice (islets’ size and staining for insulin and glucagon) but we detected a lower insulin content in the pancreas of HYP mice compared to NOD mice. These results give new insights about the role played by Akt2 in glucose homeostasis and argue for the ß cell failure being the primary event in the course of diabetes.

## Introduction

The worldwide increased prevalence of diabetes over the last decades is a serious public health problem, with dramatic consequences for healthcare systems related to considerable management costs^1^. Diabetes are chronic metabolic diseases characterised by dysfunctions in the regulation of glycaemia. Glycaemia, as part of body homeostasis, has to be tightly regulated since chronic hyperglycaemia has deleterious effects in humans such as peripheral neuropathy and nephropathy^2^.

The regulation of blood glucose level largely relies on the coordinated action of insulin and glucagon. Both hormones are released by Langerhans’ islets and exert opposite effects on glycemia. Glucagon, produced by the α cells, increases glycaemia by stimulating hepatocytes’ glucose production through neoglucogenesis and glycogenolysis^3^. In contrast, insulin, the main hypoglycaemic hormone of the body, is produced by the ß cell, in response to hyperglycaemia following food intake^4^. The production and secretion of insulin are finely tuned at multiple levels including transcription of the insulin gene, translation of the preproinsulin mRNA, folding and cleavage of the proinsulin, packaging of the insulin into granules, and granules release^5^.

Once released into the interstitial space of the pancreas, insulin reaches the blood flow. In tissues, insulin binds to the CD220 insulin receptor expressed by diverse target cells. CD220 belongs to the tyrosine-kinase receptor family. Upon ligand binding, CD220 autophosphorylates, recruits, and activates the insulin receptor substrate (IRS) proteins, which signal into the cell through multiple pathways including the Akt one^6^. The skeletal muscle is considered the principal site of insulin-stimulated glucose uptake. In the muscle cell, glucose is converted to energy or stored as glycogen. Adipose tissue and the liver also play a role in glucose uptake with subsequent storage as triglycerides in the adipocyte and glycogen in the hepatocyte.

According to pathogenesis, two major forms of diabetes predominate in patients. Type 1 diabetes, also known as juvenile diabetes, is an autoimmune disease characterised by the destruction of the ß cells by autoreactive lymphocytes. Consequently, insulin production in response to glucose becomes insufficient when the residual number of ß cells is too low^7^. In contrast, insulin resistance qualifies the most frequent type 2 diabetes, which is rather diagnosed in middle-aged adults^8^. In these patients, the uptake of glucose by peripheral tissues in response to insulin is impaired but abnormalities of the ß cells were also described more recently^9^.

Both type 1 and type 2 diabetes are thought to arise from a combination of genetic and environmental factors^10,11^. In humans, genetic association studies, including the recent genome-wide association ones, allowed the identification of multiple genetic loci influencing individual predisposition to type 1 or type 2 diabetes^12,13^. For instance, in type 1 diabetes, a strong genetic association of the disease with certain alleles of the HLA class II genes was described. In addition, the existence of neonatal diabetes mellitus as well as maturity-onset diabetes of the young, which are monogenic forms of diabetes, helps to better understand the role of specific genes in glucose homeostasis^14^. Studying animal models also greatly contributes to understanding diabetes aetiology^15^. The non-obese diabetic mouse (NOD), which also exhibits genetic susceptibility to type 1 diabetes, is a relevant spontaneous model of the disease^16^.

We describe herein the effects of a spontaneous Akt2 deficiency in a colony of NOD mice exhibiting a phenotype of early diabetes.

## Results and discussion

**Figure 1:**
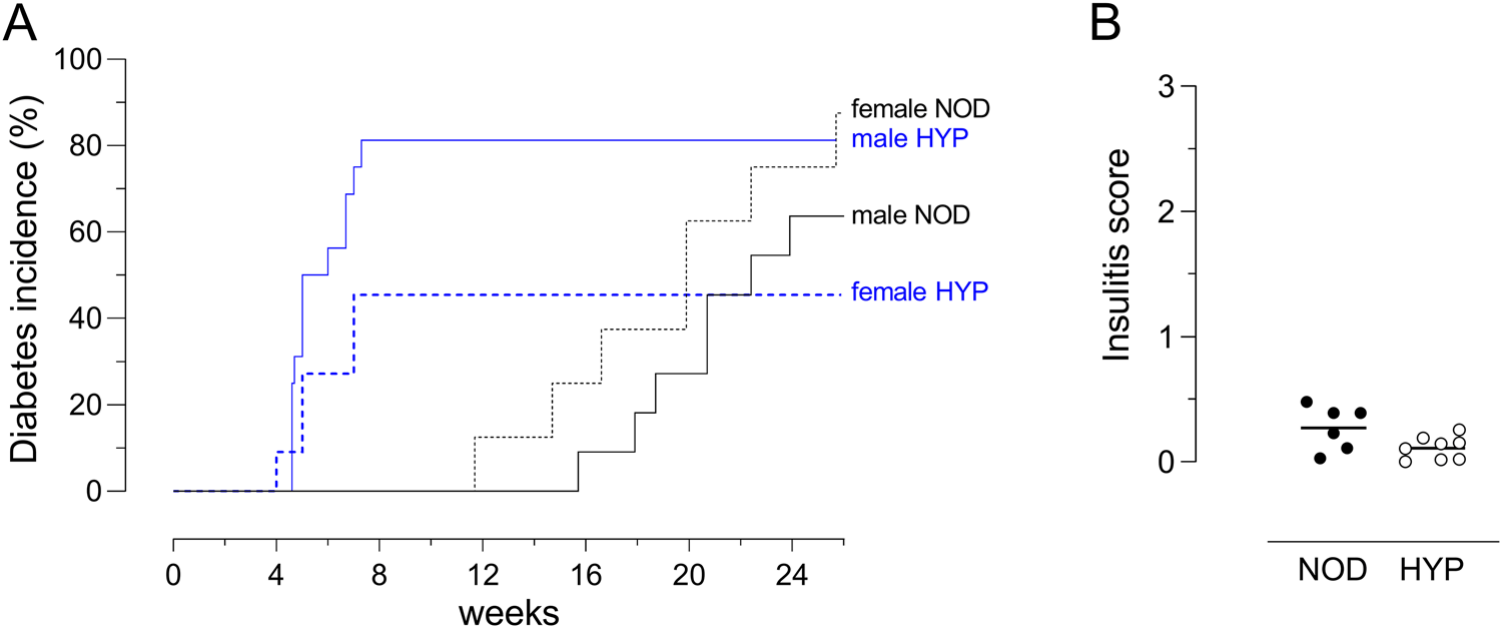
Early diabetes incidence in the HYP mice. (**A**) Glycaemia was regularly checked in male (NOD, n = 11 and HYP, n = 16) and female (NOD, n = 8 and HYP, n = 11) mice. (**B**) Insulitis was analysed in the pancreas of 7-week-old male NOD (n = 6) and HYP mice (n = 8).

A very early, hyperglycaemic phenotype was serendipitously found in males in a litter of NOD mice. This phenotype was transmittable upon an autosomal recessive pattern of inheritance, which allowed the establishment of a novel colony of mice, on the NOD background, that we called HYP. As shown in Fig. 1A, we found a higher incidence of diabetes (around 80%) in male HYP mice compared to male NOD mice, with an accelerated disease onset around the age of one month (*p* < 0.05). In females, although some HYP mice became diabetic before the age of two months, we did not detect any significant difference in the overall diabetes incidence between both strains. The analysis of insulitis in pancreatic sections of 7-week-old male HYP mice further indicated that the early hyperglycaemia was not the consequence of an acceleration of the autoimmune reaction inherent to the NOD background. Indeed, we observed similar and low infiltration of Langerhans’ islets by immune cells in both strains at this age (Fig. 1B, median insulitis score: 0.13 [0–0.26] for HYP vs 0.31 [0.03–0.48] for NOD, *p* > 0.05).

**Figure 2:**
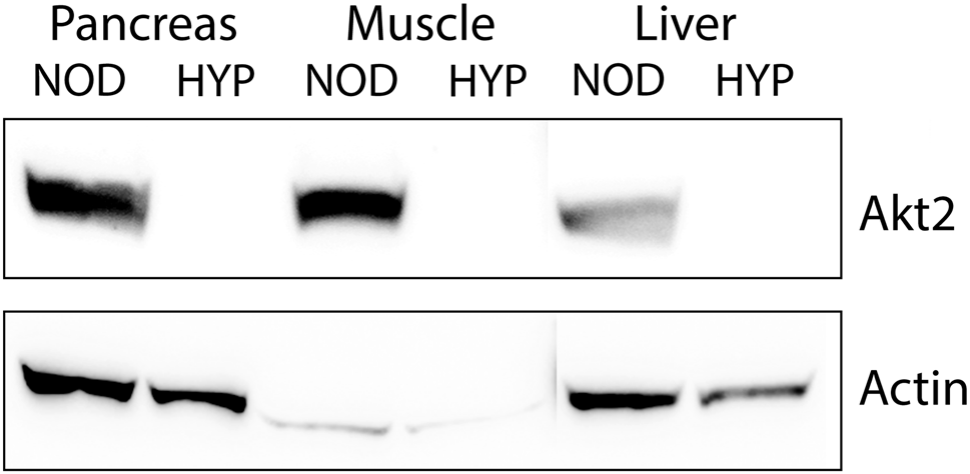
Analysis of Akt2 expression by Western blot. Protein lysates were prepared from the pancreas, liver, and muscle of NOD and HYP mice. Membranes were probed with anti-murine N-terminal Akt2 and anti-β actin antibodies. One representative Western blot is shown.

This very early onset of diabetes in the HYP mouse reminds the phenotypes grouped under the name of Neonatal Diabetes Mellitus. In humans, these forms of diabetes usually occur in infants before the age of 6 months^14^. In mice, similar phenotypes were described in the Akita and Munich models^17–19^. Both mice harbour mutations in the coding sequence of the *ins2* gene, with a substitution of a cysteine, at position 96, by a tyrosine for the Akita mouse, and, at position 95, by a serine for the Munich one. We did not find any difference in the coding sequences of the *ins1* and *ins2* genes between NOD and HYP mice. Among the 13 coding variants identified following whole genome sequencing of HYP mice compared to NOD controls, the best candidates were three frameshift variants (Akt2, Zfp69, and Tmprss13). We actually sequenced the three of them, and confirmed only the Akt2 mutation. The mutation we identified in the HYP mice consisted of a 13 bp-deletion in the exon 11 (upon 14) of the *Akt2* gene, inducing a frameshift, which inserts a premature stop codon. By Western blot, we further demonstrated the full absence of the Akt2 protein in the pancreas, muscle, and liver of HYP mice (Fig. 2 and Supplementary Fig. 1) and concluded that HYP mice were deficient for Akt2.

**Figure 3:**
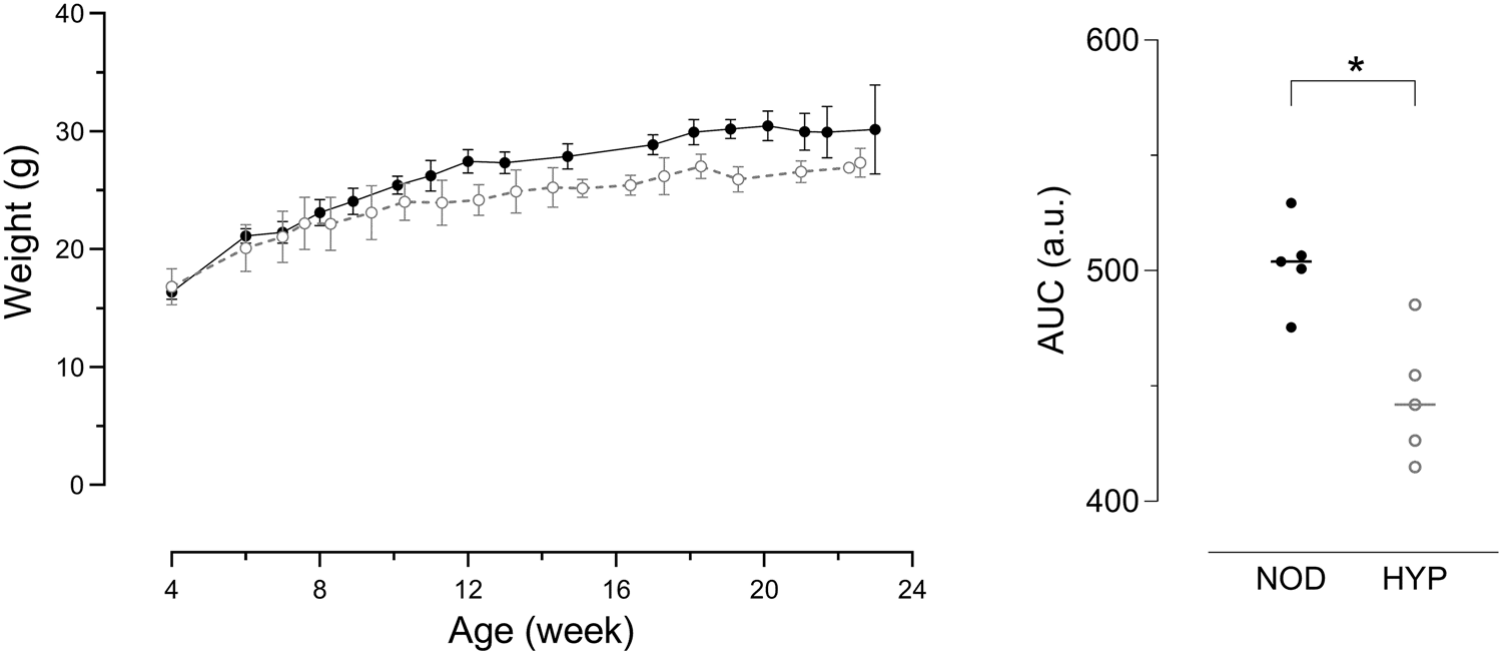
Growth curves of NOD and HYP male mice. Left, NOD (black dots) and HYP (grey dots) mice were weighed regularly until the age of 6 months (means ± SD are shown, n = 5 per group). Right, for each individual, the area under the curve (AUC) is shown.

Although male HYP mice exhibit a moderate but significant growth deficiency as compared to NOD mice (Fig. 3, p < 0.05), the hyperglycaemia induced neither abnormal weight gain nor weight loss, as classically observed in NOD mice a few weeks after diabetes onset. Garofalo et al. have also described a mild growth deficiency in the Akt2 deficient mice, which they partly attributed to progressive lipoatrophy^20^. Fischer-Posovszky et al*.* further demonstrated that Akt2 is not only involved in insulin-stimulated lipogenesis, but that it is also indispensable for the regulation of adipocyte number^21^, which may explain the growth retardation observed in the Akt2 deficient mice.

**Figure 4:**
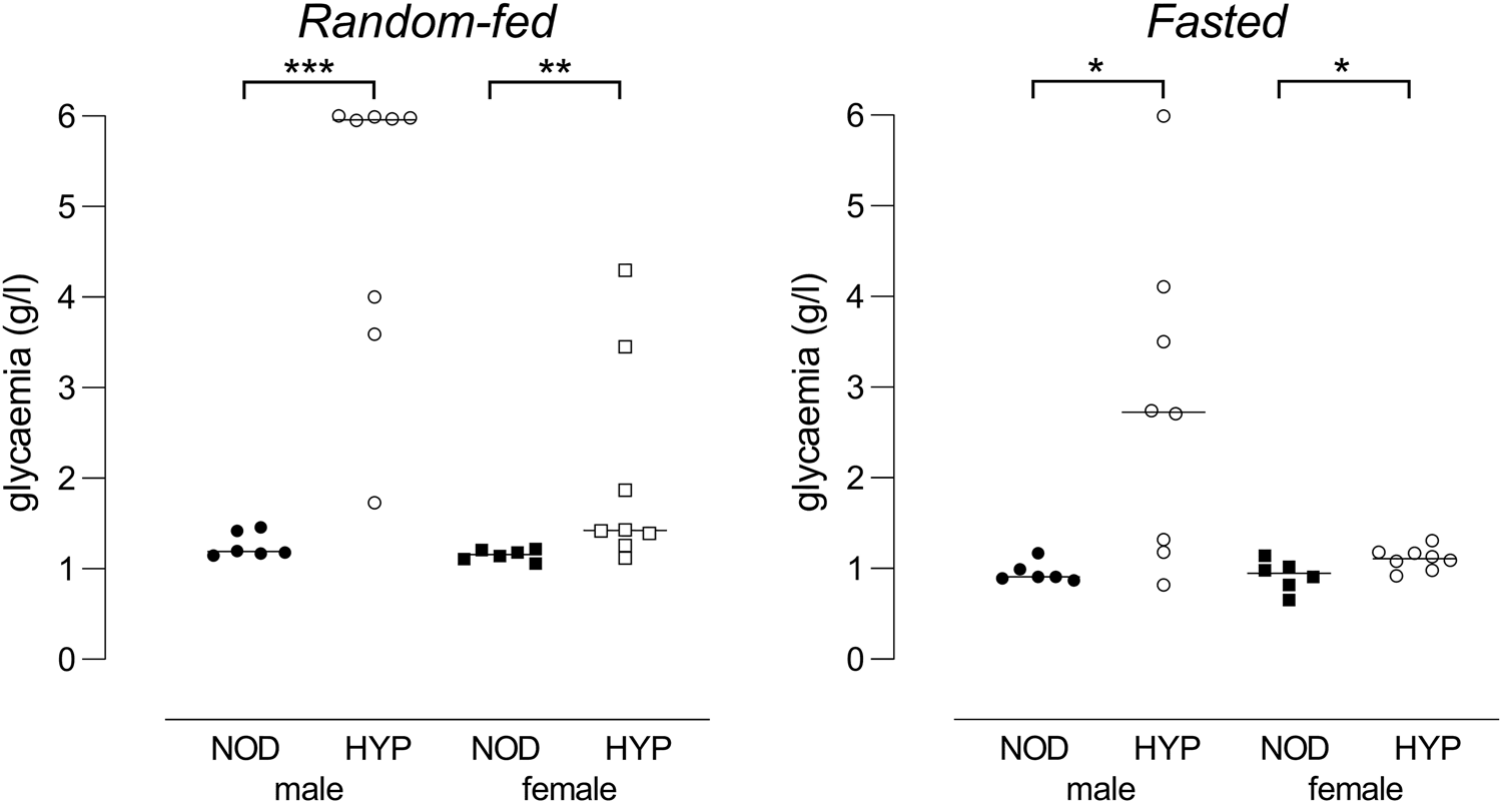
Random-fed (**A**) and fasted (**B**) glycaemia in 7-week-old NOD and HYP mice. Glycaemia was measured in NOD (n = 6/sex) and HYP (n = 8/sex) mice before and after a 4 h fasting.

Akt2 deficiency was previously demonstrated to induce hyperglycaemia in mice, with contrasted severity depending on the genetic background and the sex^20,22^. In agreement with Garofalo et al*.*^20^, we observed a stronger effect of the Akt2 deletion in male mice compared to female mice. Indeed, in accordance with the incidence curves, 7-week-old HYP females displayed milder hyperglycemia than HYP males, especially after fasting (Fig. 4). Isolated islets from male and female mice were previously shown to differ in their ability to produce insulin in response to various stimuli^23^. Additionally, recent data demonstrated that female islets exhibit a greater ability to sustain glucose-stimulated insulin production across multiple contexts^24^, which might explain the sexual dimorphism in glucose metabolism observed in Akt2 deficient mice.

**Figure 5:**
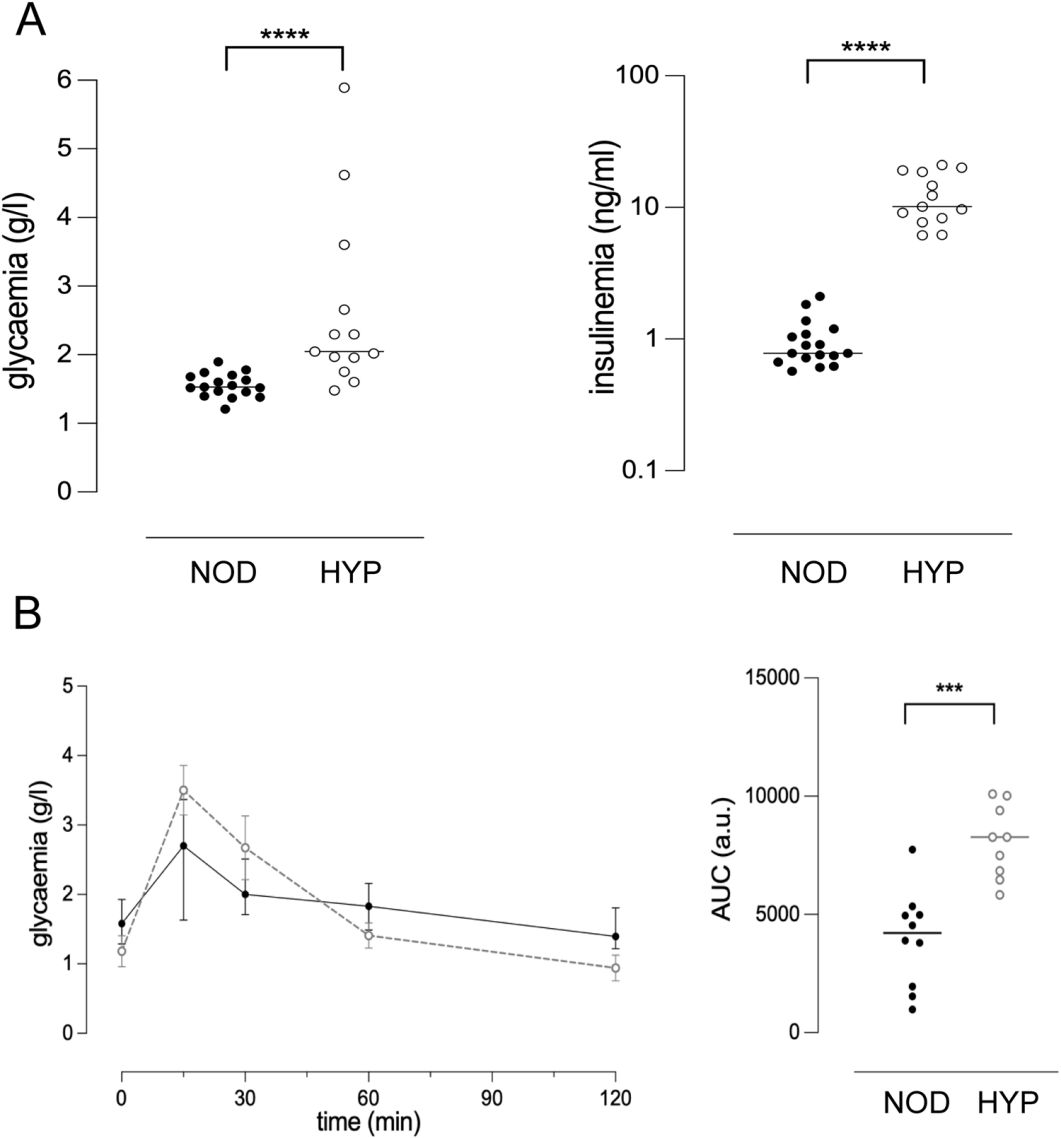
Altered glucose metabolism in one-month-old HYP male mice. (**A**) Blood glucose and insulin concentrations from random-fed males. NOD (n = 17) and HYP (n = 13) mice were issued from at least three different litters. (**B**) Glucose tolerance test. Left, means ± SD of 9–10 mice per group in two batches per strain are shown. The difference in glycaemia between NOD and HYP mice was significant at any time point (p < 0.05). Right, for each individual, the AUC was calculated using the time 0 blood glucose level as the baseline.

Focusing on male mice, we detected greater serum insulin levels in one-month-old HYP mice as compared to NOD controls (10.1 ng/ml [6.2–21.0] vs 0.8 ng/ml [0.6–2.1], *p* < 0.0001, Fig. 5A), with a milder random-fed hyperglycaemia than later on (2.05 g/l [1.48–5.89] for HYP vs 1.53 g/l [1.21–1.90] for NOD, *p* < 0.0001, Fig. 5A). Interestingly, even normoglycaemic mice exhibited hyperinsulinemia. At this young age, glucose tolerance was already significantly impaired in HYP mice (AUC: 8266 a.u. [5820–10088] vs 4219 a.u. [985–7740] for NOD mice, *p* < 0.001, Fig. 5B).

Hyperglycaemia associated with hyperinsulinemia is characteristic of insulin resistance. In Akt2 deficient mice, hallmarks of insulin resistance, such as impaired glucose uptake by muscle cells, were previously reported^20,22^. Since then, extensive literature suggested that Akt1 and Akt2 are key regulators of the insulin signalling pathway. The Akt pathway operates downstream of the insulin receptor in target cells, especially muscle cells and adipocytes, and promotes the recruitment of Glut4 at the plasma membrane to allow glucose uptake^25^. Skeletal muscle plays a major role in glucose uptake, especially in the case of hyperinsulinemia. Recently, Jaiswal et al. reported that skeletal muscle-specific deletion of Akt2 was not sufficient to induce insulin resistance and to prevent glucose uptake in mice, which argues for compensatory role for Akt1^26^. However, the co-deletion of Akt-1 and -2 in skeletal muscle had only a modest effect on glucose metabolism, suggesting the involvement of other signalling molecules in insulin resistance.

**Figure 6:**
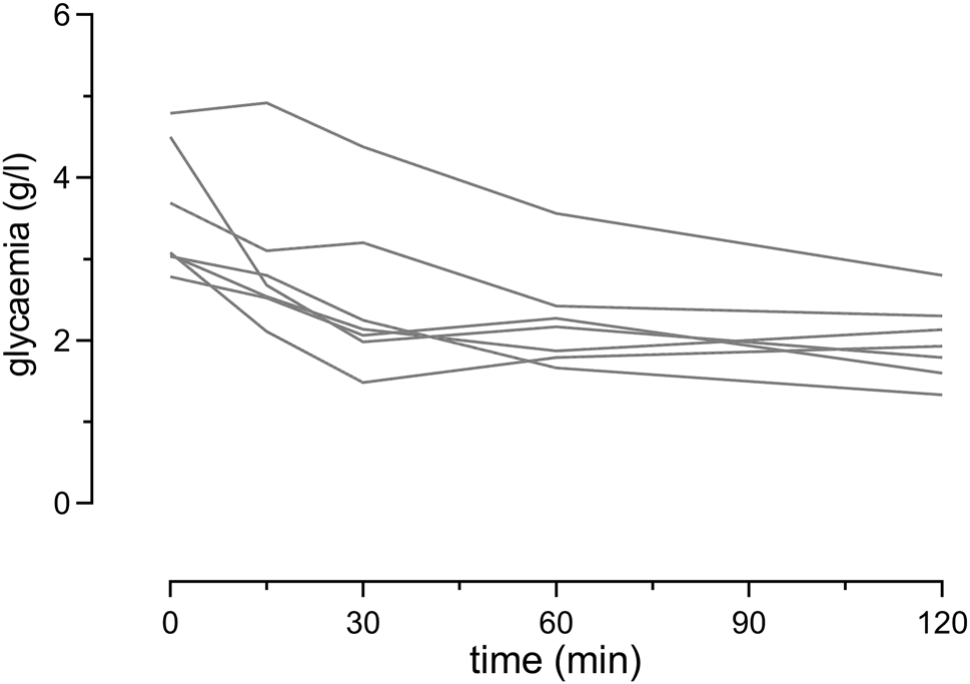
Insulin tolerance test. Glycaemia was measured just before and after the injection of insulin in 2-month-old male HYP mice (n = 7).

In type 2 diabetes, whether peripheral insulin resistance precedes impaired beta cell function is still a matter of debate^27,28^. One hypothesis is that ß cell overstimulation is not the consequence of a decreased uptake of glucose, but rather the primary failure. In this model, insulin resistance is an adaptive mechanism that prevents overstimulation of target cells. In 4-week-old HYP mice, we detected hyperinsulinemia in all individuals, including the normoglycaemic ones, suggesting that ß cell dysfunction could occur even before insulin resistance appears. Interestingly, injection of insulin in 2-month-old hyperglycaemic male HYP mice lowered glycaemia in the first 30 min (Fig. 6). This argues for the insulin resistance occurring rather after the hyperinsulinemia, although we did not explore further this point.

Although ß cells differentiate during embryogenesis, the postnatal period is also essential to the increase of the ß cell mass through proliferation, and to the maturation of ß cells, which acquire the ability to secrete insulin in response to glucose. In rodents, it was shown that the weaning period and the associated feed transition were pivotal to ß cell function^29^.

**Figure 7:**
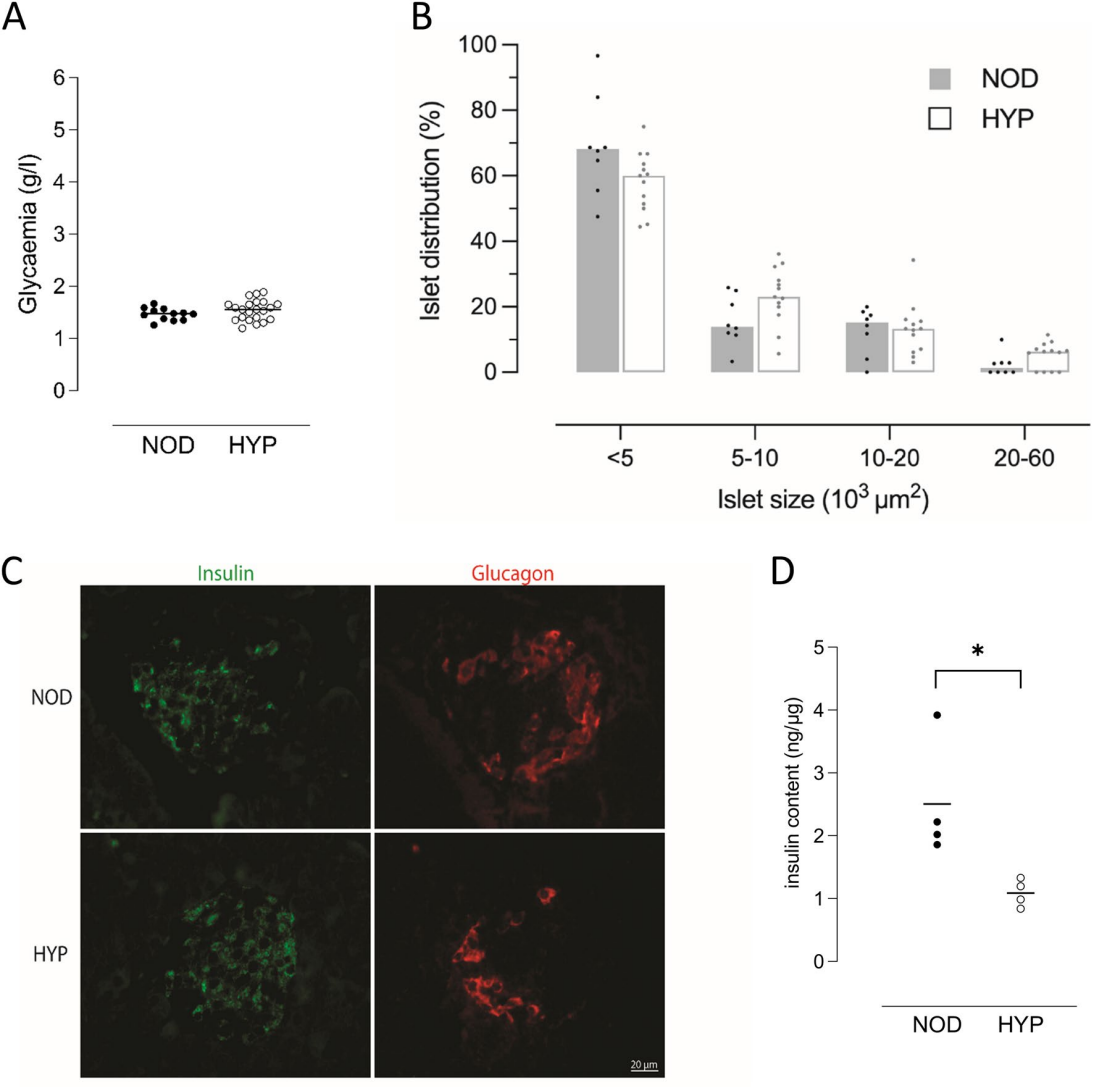
Analysis of glycaemia and pancreatic morphology in 3-week-old male HYP mice. (**A**) Random-fed glycaemia in 3-week-old NOD (n = 12) and HYP (n = 22) male mice. (**B**) Islets’ size was measured on haematoxylin and eosin-stained histological sections of pancreas from NOD (n = 8) and HYP (n = 13) male mice. (**C**) Insulin (green) and glucagon (red) staining. Representative pancreatic islets from NOD and HYP mice are shown. (**D**) Pancreatic insulin content. One experiment representative of two is shown.

We explored the pancreatic function of male HYP mice after weaning as compared to NOD controls. At the age of 3 weeks, the glycaemia of random-fed mice was similar and below 2 g/l in both strains (Fig. 7A). We did not investigate in detail the ß cell mass, but we did not find any difference using morphometry analysis. Indeed, we showed similar islet size distribution in both strains (Fig. 7B), with an islet median surface of 3598 µm^2^ [2663–5776] for HYP vs 3151 µm^2^ [777–5903] for NOD mice. In addition, both mice harbour similar distributions of α and ß cells in pancreatic islets (Fig. 7C). Interestingly, the pancreatic insulin content was significantly lower in weanling HYP mice as compared to age-matched NOD controls (1.09 ng/µg [0.84–1.33] vs 2.12 ng/µg [1.86–3.92], *p* < 0.05, Fig. 7D). Altogether, our results suggest abnormal insulin release in the HYP mice.

Although consistent with previous studies, our observations are in favour of greater consequences of the Akt2 deficiency on the NOD background. This is not surprising since alterations of the insulin release pathway in ß cells were already described in this genetic background. For example, Thurmond and collaborators reported a reduction in the expression of the calcium sensor Doc2b and the t-SNARE syntaxin-4 in the islets of non-diabetic NOD mice as compared to controls^30,31^. Both molecules are involved in the docking and fusion of the insulin granules to the plasma membrane.

Akt proteins were already shown to control insulin secretion by ß cells. Indeed, the expression of a kinase-dead mutant Akt1 in pancreatic ß cells induced hyperglycaemia associated with hypoinsulinemia in random-fed mice, while pancreatic insulin content was similar^32^. This suggests that Akt1 controls glucose-stimulated insulin secretion, which was further confirmed in vitro on murine islets^33^. Arous et al. also demonstrated that the silencing of Akt2 induced insulin release in vitro under low glucose conditions^33^. Back to our data, we confirmed in vivo that Akt2 prevents inappropriate insulin release by ß cells. Thus, the characterisation of the HYP mouse contributes to the understanding of the role played by Akt2 in ß cell function. A perfect comprehension of this role would require engineering mice with ß cell-specific Akt2 deletion.

## Materials and methods

### Ethical statement

The present study was conducted in compliance with the ARRIVE guidelines. All animal experiments were carried out in strict agreement with the EU directive 2010/063, under the authority of the French Ministry for Education and Research (APAFIS#11205-201709081535322v2) after being reviewed and approved by the French ethics committee n°6. All efforts were made to minimise animal suffering and to limit the number of experimental mice. The data presented here were collected from 197 mice).

### Animals

Control and mutant mice were kept in specific pathogen-free conditions at the Oniris rodent facility (EU 0554). They were housed in the same room in a 14 h-light 10 h-dark cycle in a controlled environment (temperature 22–25 °C, humidity 55% ± 5). Mice were fed a standard rodent diet and had ad libitum access to water.

In each experiment, mutant mice were compared to age- and sex-matched control NOD mice. The sex and the age of the mice used in each experiment are specified in the relevant sections, and the number of mice per experiment is indicated in the corresponding Figure legends.

### Glucose measurements, glucose and insulin tolerance tests

Glycaemia was measured on a drop of blood drawn from the tail vein using the One Touch Verio glucometer (LifeScan France, France). Considering the limit of detection of the instrument, glycaemia was plotted as 6 when above 6 g/l. To plot the incidence curves, mice were considered diabetic after two consecutive glycaemia above 2.5 g/l a few days apart.

When required, fasting was performed by placing mice in metabolic cages for 4 h in the mornings.

For the glucose tolerance test, 1-month-old NOD and HYP male mice were fasted for 4 h before the intraperitoneal glucose tolerance test. Glycaemia was recorded, as explained above, at time 0 and over a 2 h period following the intraperitoneal injection of glucose at a dose of 2 g/kg body weight. Insulin tolerance tests were performed in the mornings on random-fed male HYP hyperglycaemic mice: blood glucose was monitored at time 0 and over a 2 h period following insulin (Humalog, 0.75 IU/kg) injection as described above.

### DNA sequencing

Genomic DNA was isolated from ear clips of mice using the NucleoSpin Tissue Kit (Macherey–Nagel).

For whole genome sequencing, DNA libraries, from 4 HYP and 2 NOD mice, were prepared using Nextera DNA Flex Library Preparation (Illumina) following the manufacturer’s instructions, and prepared libraries were sequenced using Illumina NovaSeq 6000 (Illumina). The sequencing software ‘bcl2fastq2’ was used to demultiplex data and convert BCL files to FASTQ format for downstream alignment analyses. The sequencing reads were mapped to the B6J mouse reference genome (GRCm38) using ‘bwa’^34^, reads were marked for duplicates and recalibrated using ‘gatk4’^35^. The average per-sample depth of sequencing, obtained using ‘mosdepth’^36^, was 20X. Variants were called using HaplotypeCaller, annotated using 'bcftools csq'^37^ and then filtered using ‘jvarkit’^38^ [first for homozygous variants found at least in 3 upon 4 HYP mice (and not in the NOD mouse) and then for frameshift, missense, and inframe variants affecting protein sequences].

To confirm which gene was actually mutated in the HYP mouse, DNA from additional NOD (n = 1) and HYP mice (n = 2) was further analysed using PCR. Three pairs of primers overlapping candidate mutations in *Akt2* (forward 5′-TCTTCTCCTTCCTCCCTCAG-3′ and reverse 5′-AGGCAGACAGCTCACACACT-3′), *Zfp69* (forward 5′-ATCAAGGGAACTCCAACACA-3′ and reverse 5′-ATGGGACACAACAAGAAAGC-3′), and *Tmprss13* (forward 5′-ATTCCTCTCCCACTCACAGG-3′ and reverse 5′-CCCACTGGTGTTGCTCTAAC-3′) were used and the amplicons were sequenced (Eurofins). The *Akt2* mutation was finally further confirmed in four additional HYP mice.

### Protein lysates

Fragments of the pancreas, muscle, and liver were harvested and snap-frozen in liquid nitrogen. Tissues, placed in RIPA buffer (Sigma-Aldrich) containing 1% Protease Inhibitor cocktail (Sigma-Aldrich), were disrupted using the Retsch Mixer Mill (MM 400, Retsch) thanks to the addition of five 3 mm stainless steel balls per sample (30 Hz for 1 min, twice). After 30 min incubation at 4 °C, lysates were clarified by centrifugation (20,000 g for 15 min). Protein concentration was determined by the Micro BCA Protein assay kit (Thermo Scientific).

### Western blot analysis

Sixty micrograms of protein extract were resolved on SDS-PAGE using 4–12% Bis–Tris Plus gel in MES SDS running buffer (ThermoFisher Scientific) in reducing conditions. After transfer on nitrocellulose membranes and saturation, membranes were incubated overnight at 4 °C with a rabbit anti-Akt2 antibody directed against the N-terminal part of the protein (ab131168, Abcam), and then with HRP-conjugated anti-rabbit immunoglobulin G antibody for 1 h at room temperature (Dako). After stripping, membranes were incubated with an HRP-conjugated anti-ß actin antibody (BioLegend). Signals were visualised by enhanced chemiluminescence with West Pico Plus substrate (ThermoScientific).

### Insulin levels

To measure circulating insulin levels, blood samples were collected from the retro-orbital venous plexus of mice under gaseous anaesthesia. Serums were obtained after centrifugation of the blood at 1300*g* for 10 min and frozen at − 80 °C until use. Insulin was quantified using the Stellux mouse insulin ELISA kit (Alpco, France).

For pancreatic insulin content, the pancreas were collected from 3-week-old mice in an ice-cold ethanol-HCl solution (70–1.5%) and kept overnight at − 20 °C. Proteins were extracted according to the “Animal Models of Diabetic Complications Consortium Protocol” (http://www.diacomp.org/shared/document.aspx?id=73&docType=Protocol). Insulin was quantified as mentioned above and the insulin content was normalised to the protein one, measured by the Bradford method.

### Morphometric analysis of the islets of Langerhans and immunohistochemistry

Pancreas were formalin-fixed for 48 h and paraffin-embedded. Tissue sections were stained with haematoxylin and eosin. Islets’ size was measured on 5 µm-pancreatic sections issued from 3-week-old male mice using the Zen software (Zeiss). The insulitis scoring was performed on pancreatic sections from 7-week-old male mice (NOD, n = 6, HYP, n = 8). Briefly, individual islets were given a score of 0–3 based on the degree of lymphocytic infiltration, and an insulitis index was calculated for each mouse [adapted from Ref.^39^]. For both analyses, at least 25 islets on 2–4 sections, 200 µm apart, per pancreas were analysed.

Insulin and glucagon immunohistochemistry was performed on formalin-fixed paraffin-embedded sections of pancreas. Briefly, once deparaffinised and rehydrated, slides were heated in citrate buffer (10 mM, pH = 6) at 90–100 °C for 10 min in a microwave. Slides were incubated simultaneously with a rabbit anti-insulin antibody (Cell Signaling, clone C27C9) and a mouse anti-glucagon one (Merck, clone K79bB10). The secondary antibodies used were Alexafluor 488-conjugated anti-rabbit immunoglobulins and Alexafluor 555-conjugated anti-mouse immunoglobulins. All the morphometric analyses were conducted blind to genotype.

### Statistical analyses

Diabetes incidence was compared using the log-rank test. Other data were analysed as non-parametric ones and the Mann–Whitney test was used to compare medians in NOD vs HYP mice of the same sex, each dot representing a mouse on charts. Medians and ranges are reported in the text. For the analyses of the glucose tolerance tests means ± SD were also calculated and graphed and the area under the curves (AUC) were calculated for each individual, and an Anova followed by a Sidak’s multiple comparisons test was performed to compare means at any time point. All data were graphed using GraphPad Prism (v9.5.1). Stars on the charts indicated statistically significant differences (*p < 0.05, **p < 0.01 and ***p < 0.001, ****p < 0.0001).

### Supplementary Information

Supplementary Information 1.

Supplementary Information 2.

## Data Availability

The raw data obtained to calculate diabetes incidence, glucose tolerance and islets’ size are provided in the Supplementary Table 1. The other original data generated and analysed are included in the manuscript.
