# Supplementary material for: Spontaneous Akt2 deficiency in a colony of NOD mice exhibiting early diabetes
Source: Sci Rep. 2024 Apr 20;14:9100. doi: 10.1038/s41598-024-60021-w (PMC11032318; doi:10.1038/s41598-024-60021-w)
Supplement: Supplementary file 2 — Supplementary Information 2. [file 41598_2024_60021_MOESM2_ESM.pdf]

Supplemental Figure 1

Original images of Western blots.

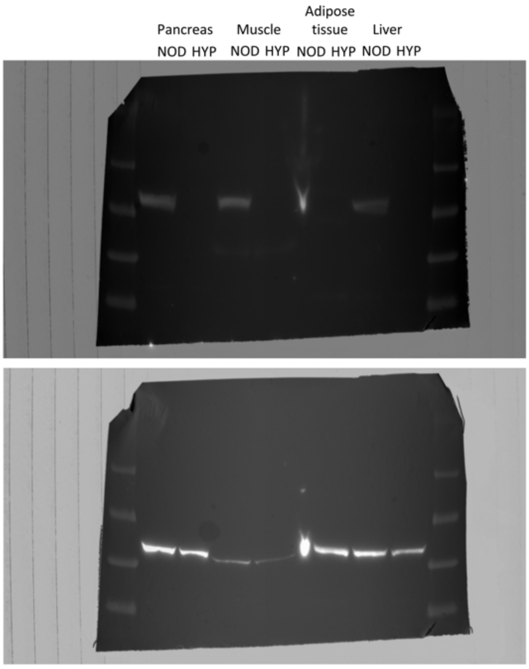

Raw data of the Western blot provided in Figure 2
Upper image: Akt2 hybridization of the membrane
Lower image: b-actin on the same membrane, after stripping.
We have cropped the adipose tissue because the migration of the NOD sample was not satisfactory.

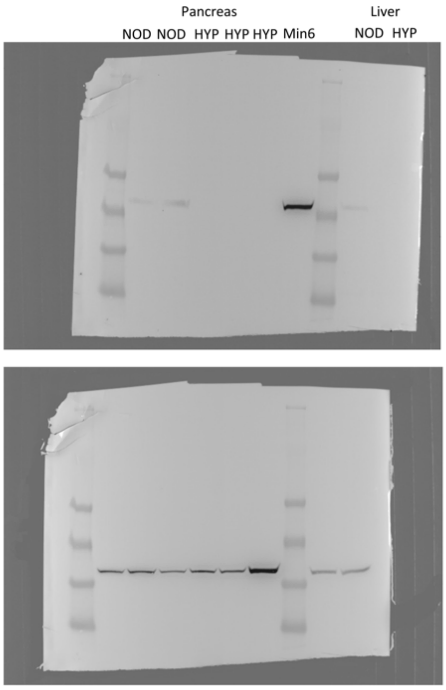

Raw data of a second Western blot
Upper image: Akt2 hybridization of a membrane loaded with other samples of pancreas and liver from NOD and HYP mice. Min6: protein extracts from a murine pancreatic cell line.
Lower image: b-actin on the same membrane after stripping

Supplemental Table 1

Raw data used to assess diabetes incidence, glucose tolerance and islets' size in NOD and HYP mice.
